# What are the limits on whale ear bone size? Non-isometric scaling of the cetacean bulla

**DOI:** 10.7717/peerj.10882

**Published:** 2021-02-05

**Authors:** Sabrina L. Groves, Carlos Mauricio Peredo, Nicholas D. Pyenson

**Affiliations:** 1 Department of Paleobiology, National Museum of Natural History, Washington, DC, USA; 2 Department of Biological Sciences, Mount Holyoke College, South Hadley, MA, USA; 3 Department of Earth and Environmental Science, University of Michigan - Ann Arbor, Ann Arbor, MI, USA; 4 Department of Marine Biology, Texas A&M University - Galveston, Galveston, TX, USA; 5 Department of Paleontology and Geology, Burke Museum of Natural History and Culture, Seattle, WA, USA

**Keywords:** Cetacean, Whale, Allometry, Bulla, Ear, Scaling, Odontocete, Mysticete, Stem cetacean, Evolution

## Abstract

The history of cetaceans demonstrates dramatic macroevolutionary changes that have aided their transformation from terrestrial to obligate aquatic mammals. Their fossil record shows extensive anatomical modifications that facilitate life in a marine environment. To better understand the constraints on this transition, we examined the physical dimensions of the bony auditory complex, in relation to body size, for both living and extinct cetaceans. We compared the dimensions of the tympanic bulla, a conch-shaped ear bone unique to cetaceans, with bizygomatic width—a proxy for cetacean body size. Our results demonstrate that cetacean ears scale non-isometrically with body size, with about 70% of variation explained by increases in bizygomatic width. Our results, which encompass the breadth of the whale fossil record, size diversity, and taxonomic distribution, suggest that functional auditory capacity is constrained by congruent factors related to cranial morphology, as opposed to allometrically scaling with body size.

## Introduction

The evolutionary history of cetaceans exhibits dramatic transformations that have facilitated their ecological transition from a terrestrial to an obligate marine lifestyle (Pyenson, 2017; Zimmer, 2011). The cetacean fossil record shows extensive anatomical modifications that allowed for this transition by facilitating communication and navigation underwater. This adaptation to life in the water, from terrestrial ancestry, required surmounting or accommodating physical constraints to the functional challenges for hearing (Nummela et al., 2007; Ketten, 1994). Previous studies have documented allometric patterns associated with precocial growth in the ear bones (i.e., tympanoperiotic complex) of living cetaceans, demonstrating that extant cetacean ontogeny is, at least partially, driven by acoustic ecology (Lancaster et al., 2015; Yamato & Pyenson, 2015; Ekdale & Racicot, 2015; Thean, Kardjilov & Asher, 2017). This study seeks to understand the allometry of cetacean ear bones across their evolutionary history to elucidate the extent to which acoustic ecology constrains variability in tympanic bulla morphology.

The cetacean auditory system has undergone dramatic modifications associated with at least three major shifts throughout cetacean evolutionary history: (1) the land-to-sea transition; (2) ultrasonic hearing for echolocation; and (3) infrasonic hearing in mysticetes (Ritsche et al. 2018; Thean, Kardjilov & Asher, 2017; Thewissen & Williams, 2002; Spoor & Thewissen, 2008; Thewissen et al., 2001; Fleischer, 1976; Schevill & McBride, 1953). Throughout these changes, cetaceans have maintained a unique auditory structure: the pachyosteoslerotic tympanic bulla. The tympanic bulla’s large, dense, conch-shaped structure works with the mandibles and soft tissues of the inner ear (e.g., inside the periotic) to detect and isolate sound (Luo & Gingerich, 1999; Cozzi et al., 2015; McCormick et al., 1970). The bulla combines with the periotic to form the tympanoperiotic complex (Mead & Fordyce, 2009). The tympanoperiotic complex is highly diagnostic for taxonomic and phylogenetic research (Ekdale, Berta & Demere, 2011; Ekdale & Racicot, 2015), and it is readily preserved in the fossil record, providing a marker of acoustic evolution (Churchill et al., 2016; Park, Fitzgerald & Evans, 2016; Park et al., 2019; Mourlam & Orliac, 2017; Racicot, Darroch & Kohno, 2018; Racicot et al., 2019). Thus, this anatomical unit is useful for studying allometric patterns in cetacean evolutionary history.

Here, we use a comparative dataset of cetacean tympanic bullae, generated from museum specimens and the published literature, spanning the full range of cetacean body size, to test the extent to which body size drives tympanic bulla size. Previous work has shown that some inner ear structures (specifically the bony labyrinth) are strongly correlated with body mass (Ekdale & Racicot, 2015; Racicot et al., 2016). However, biological systems rarely scale isometrically, and modern whales are seemingly approaching an upper limit on body size (Slater, Goldbogen & Pyenson, 2017; Goldbogen et al., 2019; Gearty, McClain & Payne, 2018), suggesting osteological and/or ecological constraints on scaling. Our study demonstrates that bullae become proportionally smaller as body size increases. The dataset relies on accessible, low-cost measurement techniques, and includes fossils spanning all of cetacean evolutionary history, including the earliest semi-aquatic stem cetaceans, and major ecological transitions (Pyenson, 2017). We demonstrate that the scaling of tympanic bullae is positively allometric, non-isometric, and smaller than anticipated at the largest body sizes.

## Materials and Methods

### Anatomical measurements

**Figure 1:**
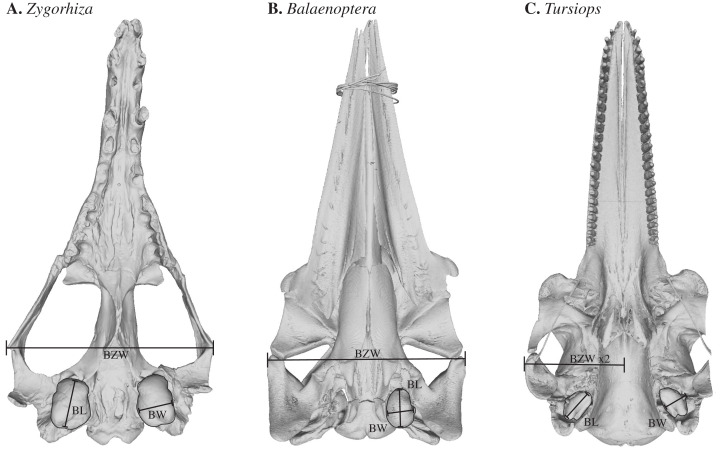
3D models of sample cetacean skulls illustrating the measurements collected for this study including (A) a stem cetacean (*Zygorhiza*, USNM PAL 11962), (B) a mysticete (*Balaenoptera*, USNM VZ 593554), and (C) an odontocete (*Tursiops* USNM VZ 550969). Specimens are scaled to the same condylobasal length. BZW: Bizygomatic width, measured as the maximum distance across the zygomatic processes of the squamosals or estimated by doubling the measurement to the midline. BL: tympanic bulla length measured along its longest anteroposterior axis following the orientation guidelines of Mead & Fordyce (2009). BW: tympanic bulla width measured along its widest transverse axis following the orientation guidelines of Mead & Fordyce (2009).

We measured the bizygomatic width (BZW), tympanic bulla length (BL), and tympanic bulla width (BW) of cetacean skulls using handheld calipers (±1 mm). Bizygomatic width was defined as the maximum distance between the lateral edges of the zygomatic processes and was used as a proxy for cetacean body size (Pyenson & Sponberg, 2011). In the case of incomplete skulls, the bizygomatic width was measured from the lateral edge of one zygomatic process to the midline and doubled. BL was measured in the dorsal and lateral views from the outer posterior prominence to the edge of the involucral ridge following previous authors and as documented by Tsai & Fordyce (2015) and references therein. Bulla width was measured in ventral views from the mallear ridge to the involucrum following Tsai & Fordyce (2015) and Tanaka, Ando & Sawamura (2018) (Fig. 1). Where possible, we measured both the right and left bulla and used the mean value in this study. Only complete and intact specimens were included in the final dataset. Other studies have used the periotic, specifically inner ear structures such as the spiral cochlea and the bony labyrinth, to test for changes in acoustic ecology through whale evolutionary history. Here, we elect to focus on the tympanic bulla because it is an external structure that can be measured with minimal resource allocations and because tympanic bullae preserve readily in the fossil record, making it easier to amass a large dataset that can be easily replicated.

### Data acquisition and taxonomic selection

We measured the bizygomatic width, bulla length, and bulla width for specimens that preserve both skulls and at least one complete tympanic bulla. Our data set includes fossil cetaceans from the UMMP and USNM; we then supplemented this dataset with additional measurements from published specimens from the literature. Juvenile and subadult specimens were excluded as examining ontogenetic growth is beyond the scope of this study. The final dataset (Table S1) includes 267 representatives of nearly every known cetacean taxon (*n* = 135) with pairable bizygomatic widths and tympanic bulla.

### Phylogenetic analysis

To test for potential phylogenetic signal, we constructed a composite tree using previously established phylogenetic relationships and their heuristic searches with accepted support values (Lambert, Bianucci & De Muizon, 2017; Tanaka & Fordyce, 2017; Marx & Fordyce, 2015; Peredo & Uhen, 2016; Gatesy et al., 2013; O’Leary, 2001). The composite matrix, constructed in MESQUITE 3.6 (Maddison & Maddison, 2018), included three new continuous characters: BZW, BL, and BW. Phylogenetic Independent Contrasts (PICs) correlated continuous size variable traits with corresponding taxa using non-transformed data in PDTREE. Branch lengths were set to 1.0 and colors were allocated by character value (Pyenson, Goldbogen & Shadwick, 2013). PIC axes were set as follows: Y-the character for exploration (**|**BL:BZW**|**) and X-the tree character ($\sqrt {\; \Sigma {\rm\rho} \left( {X,Y} \right)}$, the square root of the sum of the correlated branch lengths). To assess the phylogenetic underpinnings of non-isometric scaling relationships, we regressed the PICs of the continuous character traits and mapped them back onto the original composite tree (Garland & Ives, 2000; Pyenson, Goldbogen & Shadwick, 2013). The dataset exhibited a normal distribution and character trait ranges were spread across families.

## Results

### Allometry of cetacean tympanic bullae

**Figure 2:**
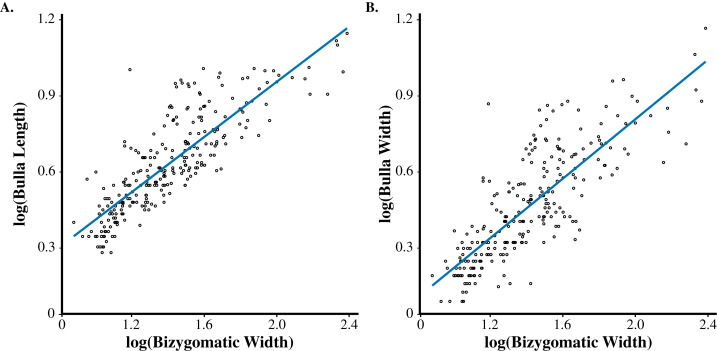
Log-transformed bivariate plot demonstrating allometric changes in bulla size and bizygomatic width. (A) Tympanic bulla length vs bizygomatic width. (B) Tympanic bulla width vs bizygomatic width. Black dots represent specimens from the amalgamate dataset. Colored lines represent linear regressions. See text for statistical results.

Scaling relationships of tympanic bulla length (Fig. 2A slope = 0.5488×, *R*^2^ = 0.7055) and bulla width (Fig. 2B slope = 0.5644×, *R*^2^ = 0.6824) vs bizygomatic width were positively allometric (Fig. 2). This trend suggests that body size is the predominant correlate influencing ear size, with roughly 70% of the bullae dimensional variation being explained by changes in body size. We used log-transformed plots to display linear regressions across the sample, allowing size extremes to be shown with minimal axis compression (Fig. 2). The smallest cetaceans (e.g., *Cephalorhynchus hectori*, *Pontoporia blainvillei*, and *Phocoena phocoena*) had bullae that were about twice as long as they were wide (BL:BW 1.7–2.2). Conversely, the largest cetaceans (e.g., *Eubalaena glacialis, Megaptera novaeangliae*, *Balaenoptera physalus*) exhibited bullae nearly as wide as they were long (BL:BW 1.1–1.7). At smaller body sizes (BZW < 185 mm), the tympanic bulla length was consistently 15–41% of bizygomatic width. However, at larger body sizes (BZW > 407 mm) bulla length was closer to 10% and as low as 4% of bizygomatic width in some specimens of *Megaptera novaeangliae* and *Balaenoptera physalus*, indicating that tympanic bullae are proportionally smaller at the largest body sizes.

**Figure 3:**
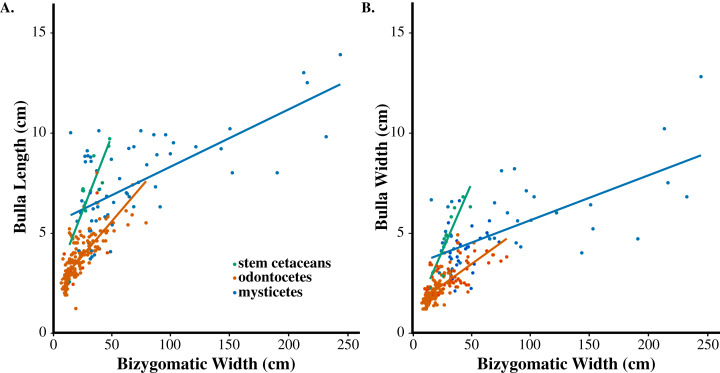
Allometric relationships of stem cetaceans, odontocetes, and mysticetes. (A) Tympanic bulla length (BL) vs bizygomatic width (BZW). (B) Tympanic bulla width (BW) vs bizygomatic width (BZW). Green circles ****represent stem cetaceans, red ****correspond with odontocetes, and blue ****indicate mysticetes. Colored lines represent linear regressions by group.

The patterns observed in the cumulative dataset remain consistent within taxonomic groupings (stem cetaceans, odontocetes, and mysticetes). Larger body sizes were correlated with longer tympanic bulla in all three groups (Fig. 3): stem cetaceans (slope = 0.1626×, *R*^2^ = 0.7166), mysticetes (slope = 0.0248×, *R*^2^ = 0.4635), and odontocetes (slope = 0.049×, *R*^2^ = 0.5868). Similar patterns were observed for body size and tympanic bulla width in stem cetaceans (slope= 0.0034×, *R*^2^ = 0.7719), mysticetes (slope= 0.0217×, *R*^2^ = 0.4100), and odontocetes (slope = 0.04×, *R*^2^ = 0.5293).

Within groups, our data demonstrated insignificant linear growth trajectories, with stem cetaceans and odontocetes constrained to the left side of the graph likely as a result of their smaller body sizes, and mysticetes occupying a wide range of ear and body sizes (Figs. 2 and 3). As a paraphyletic group, stem cetaceans resemble the tympanic bullae size and proportions of odontocetes despite larger body sizes comparable to those of smaller mysticetes (Fig. 3). The composite dataset includes a diverse assortment of bulla and bizygomatic sizes.

Tympanic bullae and bizygomatic width seemingly conform to the same scaling coefficient, regardless of taxonomic grouping (Figs. 2 and 3). Our phylogenetic independent contrasts (PIC) yielded no genus-level clustering in both branch proximity and corresponding character traits, indicating that tympanic bulla size is not governed by phylogeny (Fig. S1).

## Discussion

### Scaling and function

Tympanic bullae play a fundamental role in cetaceans’ abilities to navigate, communicate, and feed within aquatic systems. Our results demonstrate that cetacean bulla dimensions increase in a positively allometric pattern irrespective of taxonomic identity or phylogenetic history. Nonetheless, the largest cetaceans (mysticetes) exhibit disproportionately small tympanic bullae, while small-bodied cetaceans (e.g., *Pontoporia, Platanista*, phocoenids, and extinct odontocetes such as *Olympicetus* and *Echovenator*) exhibit particularly large ears for their body sizes (Fig. 3). These small-bodied odontocetes all retain proportionately large tympanoperiotic complexes, possibly hinting at a lower limit for cetacean bulla size. Notably, the largest cetaceans are all extant (Rosel et al., 2020; Pyenson & Sponberg, 2011; Pyenson & Vermeij, 2016; Slater, Goldbogen & Pyenson, 2017). Whale body size persists near a lower bound for much of their evolutionary history and only reached extreme gigantism during the Plio-Pleistocene (Slater, Goldbogen & Pyenson, 2017). Such departures from linearity suggest that functional auditory capacity is not based on proportional congruences, but may instead be constrained by functional or biological auditory limits.

One such constraint may be osteological: the tympanic bulla functions by acoustically isolating the hearing apparatus from the rest of the skull (Luo & Gingerich, 1999; Nummela et al., 2004; Cozzi et al., 2015) and it remains unclear how acoustic isolation functions at proportionally larger body sizes. Another potential limitation may be ecological. The pachyosteosclerotic bulla enhances the reception of sound underwater, and may therefore be bound within a functional size range with upper and/or lower limits of effectiveness. This constraint is likely true for echolocating odontocetes, which rely on high frequency sounds not just for communication, but for navigation and feeding as well (Ketten, 1994). Future research is needed to determine how bulla size influences sound reception underwater. Finally, cetaceans often exhibit paedomorphic ear bone morphology at birth (Cozzi et al., 2015; Yamato & Pyenson, 2015), suggesting that future work examining changes in allometry across whale ontogeny may reveal developmental constraints on ear bone scaling. Such studies would necessarily focus on extant sampling, as developmental series are mostly lacking from the fossil record of cetaceans.

### Evolutionary patterns

Cetaceans underwent major morphological transformations associated with an increasingly marine lifestyle, but our results demonstrate that tympanic bulla allometry remains relatively unchanged throughout 50 million years of cetacean evolutionary history. Stem cetaceans maintain a stronger consistent relationship between tympanic bulla dimensions and body size than either of the crown groups (Fig. 3). This pattern may hold because stem cetaceans exhibit small and medium body sizes overall, but generally not the gigantism observed in extant mysticetes (Fig. 3). Despite innovations that involve hearing, such as ultrasonic echolocation in odontocetes and extreme gigantism in mysticetes, neither extant lineage differs markedly from stem cetaceans in terms of tympanic bullae dimensions and scaling. This result is noteworthy given their seemingly disparate ecologies and suggests little functional selection on tympanic bulla dimensions. Instead, bulla dimensions converge around a common form. The consistency of tympanic bulla dimensions across the land-to-sea transition, even in stem cetaceans, reinforces the hypothesis that even the earliest cetaceans already had aquatic-adapted tympanic bullae (Luo & Gingerich, 1999; Nummela et al., 2004).

Notably, while our study examines the relationship between tympanic bullae size and body size, it does not directly test whether changes in tympanic bulla size are driven by ecological factors. Future studies might test specific ecological factors as potential drivers of bulla size to help elucidate the relationship between ear size and functional ecology. For example, it remains unclear whether bullae can reach substantially larger sizes, or if the observed values in extant whales represent an upper limit, as seems to be the case for body size (Slater, Goldbogen & Pyenson, 2017). Further study in this regard will reveal to what extent tympanic bulla size and shape are restrained by functional ecology. Recent authors have begun to elucidate the specific mechanism for infrasonic hearing in mysticetes (Park et al., 2017; Ekdale & Racicot, 2015), though it remains overall less understood than ultrasonic hearing in odontocetes. Consequently, future work in this area has the potential to inform a potential relationship between mysticete hearing and mysticete gigantism.

## Supplemental Information

10.7717/peerj.10882/supp-1 Supplemental Information 1 The amalgamated phylogenetic tree used to compare stem cetaceans, mysticetes, and odontocetes for the PIC. Branches and nodes are colored by their character trait value, bulla length: bizygomatic width.

10.7717/peerj.10882/supp-2 Supplemental Information 2 The amalgamated phylogenetic tree used to compare stem cetaceans, mysticetes, and odontocetes for the PIC. Branches and nodes are colored by their character trait value, bulla length: bizygomatic width.

10.7717/peerj.10882/supp-3 Supplemental Information 3 Raw data. Measurements of bulla length, bulla width, and bizygomatic width.

## Institutional abbreviations

UMMP: University of Michigan Museum of Paleontology, Ann Arbor, Michigan, USA
USNM: Departments of Paleobiology and Vertebrate Zoology (Division of Mammals), National Museum of Natural History, Smithsonian Institution, Washington, District of Columbia, USA

## Additional Information and Declarations

### Data Availability

The following information was supplied regarding data availability: Raw data is available in the Supplemental Files.
