# Supplementary figures and images for: What are the limits on whale ear bone size? Non-isometric scaling of the cetacean bulla
Source: PeerJ. 2021 Feb 5;9:e10882. doi: 10.7717/peerj.10882 (PMC7869665; doi:10.7717/peerj.10882)

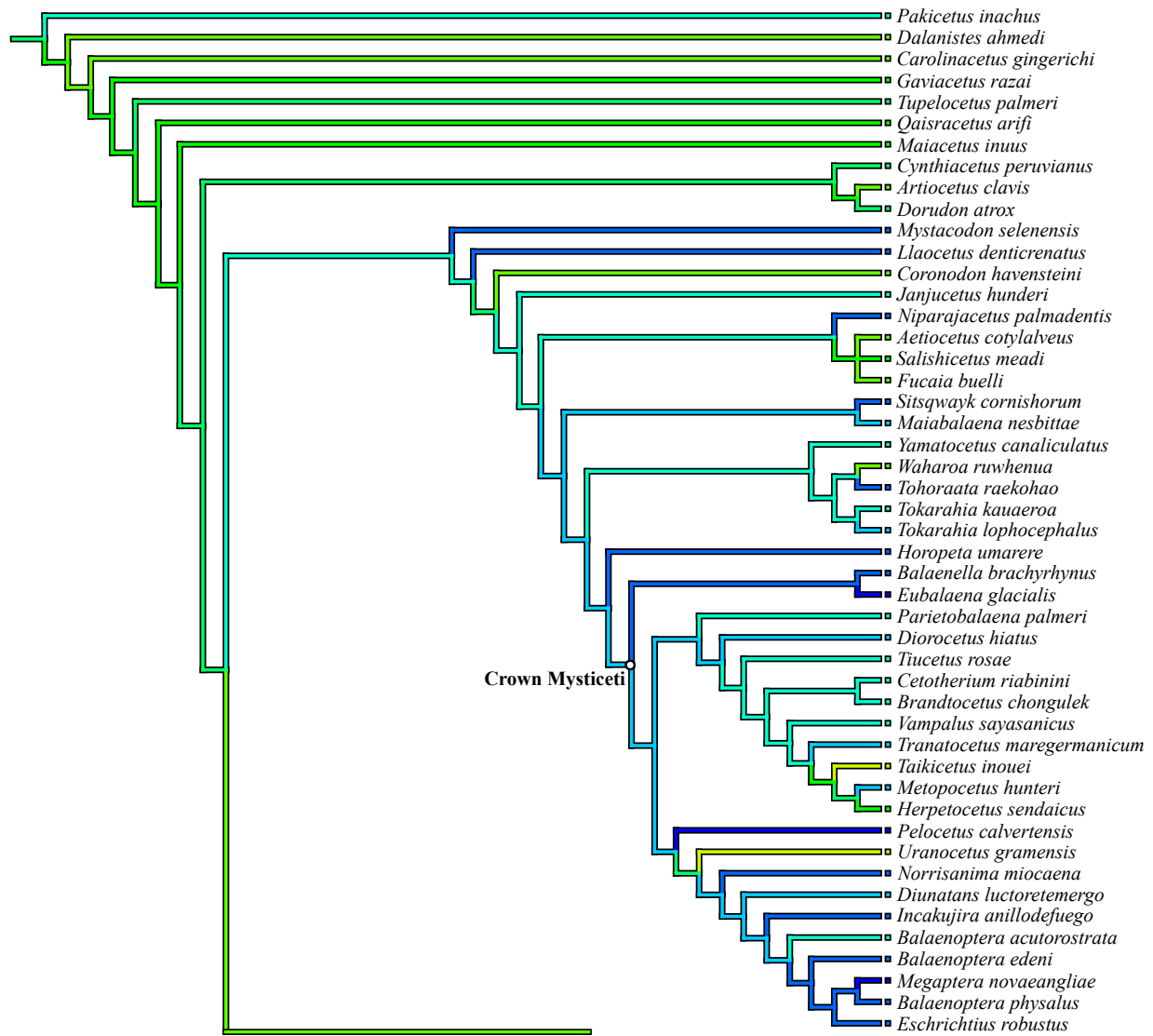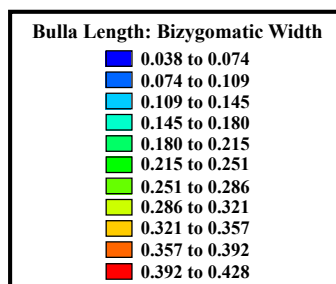

Supplement: Supplemental Information 1 — Branches and nodes are colored by their character trait value, bulla length: bizygomatic width. [file peerj-09-10882-s001.pdf]

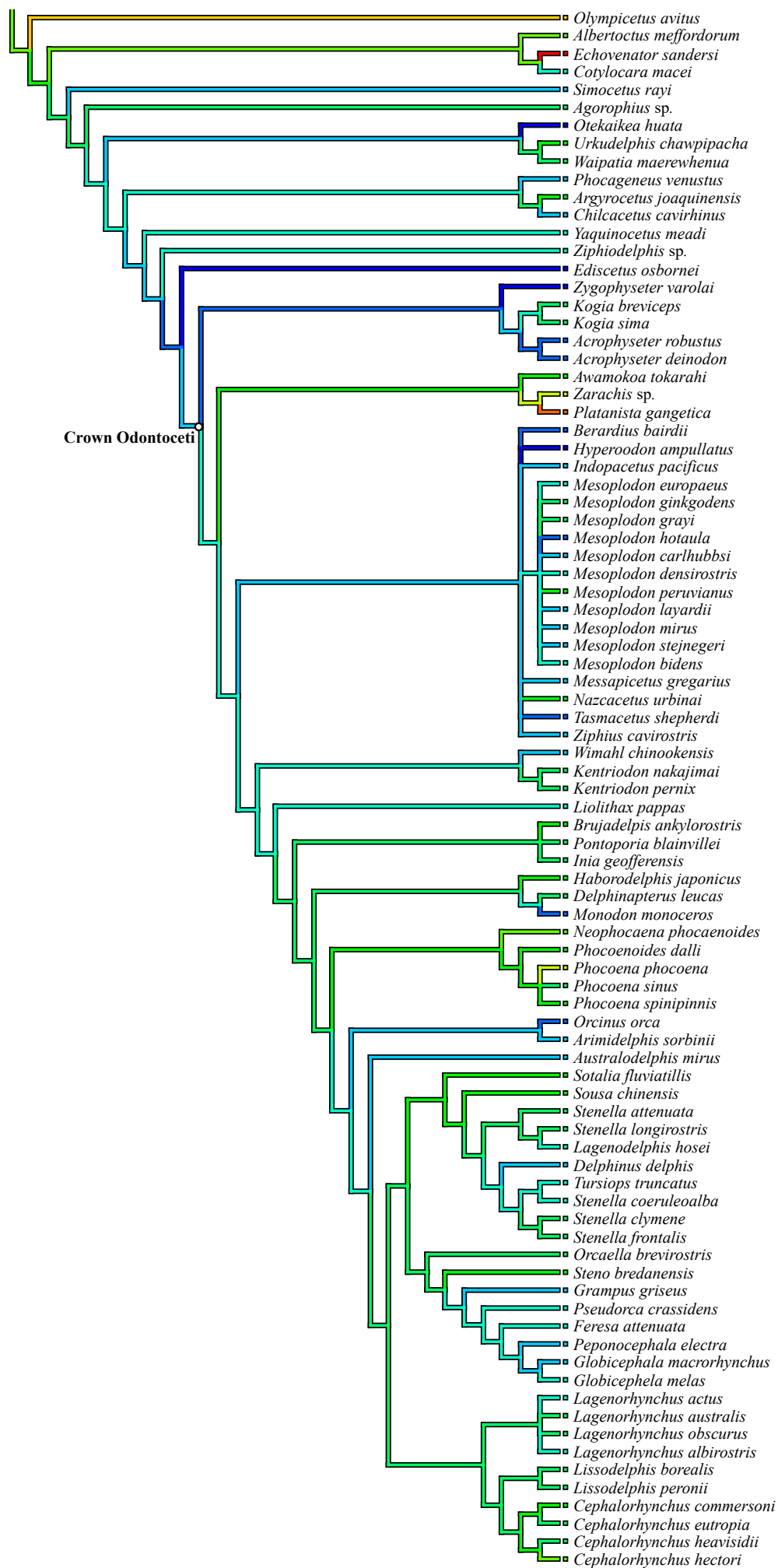

Supplement: Supplemental Information 2 — Branches and nodes are colored by their character trait value, bulla length: bizygomatic width. [file peerj-09-10882-s002.pdf]
